# Leveraging Virtual Reality and Augmented Reality to Combat Chronic Pain in Youth: Position Paper From the Interdisciplinary Network on Virtual and Augmented Technologies for Pain Management

**DOI:** 10.2196/25916

**Published:** 2021-04-26

**Authors:** Deirdre E Logan, Laura E Simons, Thomas J Caruso, Jeffrey I Gold, Walter Greenleaf, Anya Griffin, Christopher D King, Maria Menendez, Vanessa A Olbrecht, Samuel Rodriguez, Megan Silvia, Jennifer N Stinson, Ellen Wang, Sara E Williams, Luke Wilson

**Affiliations:** 1 Department of Anesthesiology, Critical Care and Pain Medicine Boston Children's Hospital Boston, MA United States; 2 Department of Psychiatry Harvard Medical School Boston, MA United States; 3 Department of Anesthesiology, Perioperative, and Pain Medicine Stanford University School of Medicine Palo Alto, CA United States; 4 Lucile Packard Children’s Hospital Stanford Stanford University Stanford, CA United States; 5 Department of Anesthesiology Keck School of Medicine University of Southern California Los Angeles, CA United States; 6 The Saban Research Institute Children's Hospital Los Angeles Los Angeles, CA United States; 7 Department of Pediatrics Keck School of Medicine University of Southern California Los Angeles, CA United States; 8 Department of Psychiatry and Behavioral Sciences Keck School of Medicine University of Southern California Los Angeles, CA United States; 9 Stanford University Stanford, CA United States; 10 Department of Pediatrics University of Cincinnati College of Medicine Cincinnati, OH United States; 11 Division of Behavioral Medicine and Clinical Psychology Cincinnati Children's Hospital Medical Center Cincinnati, OH United States; 12 Center for Understanding Pediatric Pain (CUPP) Cincinnati Children’s Hospital Medical Center Cincinnati, OH United States; 13 Department of Anesthesiology Cincinnati Children's Hospital Medical Center Cincinnati, OH United States; 14 Department of Physical Therapy and Occupational Therapy Services Boston Children's Hospital Boston, MA United States; 15 Pediatric Pain Rehabilitation Center Boston Children's Hospital Boston, MA United States; 16 Hospital for Sick Children Toronto, ON Canada; 17 Mighty Immersion, Inc. New York, NY United States

**Keywords:** virtual reality, pediatric, chronic pain

## Abstract

**Background:**

Virtual reality (VR) and augmented reality (AR) interventions are emerging as promising tools in the treatment of pediatric chronic pain conditions. However, in this young field, there is little consensus to guide the process of engaging in the development and evaluation of targeted VR-based interventions.

**Objective:**

The INOVATE-Pain (Interdisciplinary Network on Virtual and Augmented Technologies for Pain management) consortium aims to advance the field of VR for pediatric chronic pain rehabilitation by providing guidance for best practices in the design, evaluation, and dissemination of VR-based interventions targeting this population.

**Methods:**

An interdisciplinary meeting of 16 academics, clinicians, industry partners, and philanthropy partners was held in January 2020.

**Results:**

Reviewing the state of the field, the consortium identified important directions for research-driven innovation in VR and AR clinical care, highlighted key opportunities and challenges facing the field, and established a consensus on best methodological practices to adopt in future efforts to advance the research and practice of VR and AR in pediatric pain. The consortium also identified important next steps to undertake to continue to advance the work in this promising new area of digital health pain interventions.

**Conclusions:**

To realize the promise of this realm of innovation, key ingredients for success include productive partnerships among industry, academic, and clinical stakeholders; a uniform set of outcome domains and measures for standardized evaluation; and widespread access to the latest opportunities, tools, and resources. The INOVATE-Pain collaborative hopes to promote the creation, rigorous yet efficient evaluation, and dissemination of innovative VR-based interventions to reduce pain and improve quality of life for children.

## Introduction

### Background

The integration of digital health technologies into the treatment of acute and chronic pain has accelerated in the last decade. Digital health has the potential to increase patient engagement, reduce access barriers, and enhance patient-centered care, with the central goal of alleviating pain and disability in patients with chronic pain. Among these novel technologies are virtual reality (VR) and augmented reality (AR), which allow users to engage completely (VR) or partially (AR) in immersive and interactive digital experiences. VR and AR-based interventions have been found to be effective in reducing acute pain from medical procedures associated with transient pain (eg, distraction) among adults [1-3] and youth [1,4-6]. In this context, the effectiveness of VR arises from its ability to provide multisensorial engagement that can compete with pain signaling while also eliciting enjoyment and decreasing anxiety and negative mood [7]. Youth are particularly well suited to benefit from VR and AR-based pain interventions given their facility with technology and the ease with which they can engage in imaginative experiences [8].

Applying VR and AR interventions to persistent or chronic pain treatment often provides immersive experiences that go beyond redirecting attention away from a discrete painful stimulus to include physical, cognitive, and affective therapeutic targets [9]. There is growing recognition of the potential benefits of VR and AR for persistent pain, but our understanding of the mechanisms of effect and efforts to maximize the potential of these approaches for patients with persistent pain is just beginning to emerge, with those targeting youth with chronic pain being rare. To address this gap, we convened a group of pediatric pain experts to review the state of the field, identify important directions for research-driven innovation in VR and AR clinical care, and establish a consensus on best methodological practices to adopt in future efforts to advance the research and practice of VR and AR in pediatric pain. This paper summarizes the processes and outcomes of this meeting. Specifically, we outline key gaps in the field, examine models of collaboration to advance the development and evaluation of targeted VR technology, and offer recommendations for best practices for future efforts to advance the study and use of VR and AR in the treatment of pediatric chronic pain.

VR and AR interventions for the acute pain context rely primarily on VR’s immersive capabilities to distract from the discomfort of the pain experience. In the context of persistent pain, intervention targets must be broad in scope (eg, address pain-related functioning) and enduring in their effects (eg, providing a sustained reduction in pain and/or improvement in functioning lasting beyond the time spent in the VR environment). In a recent systematic review of VR and AR for pain in adults (which included chronic pain cohorts), Mallari et al [2] concluded that VR appears to reduce chronic pain intensity while VR exposure is ongoing, but there is weak evidence for any lasting post-VR exposure effects on pain intensity with existing protocols. Of 10 studies on VR and AR for chronic pain included in the review, only 2 studies [10,11] expanded beyond a focus on pain intensity to measure functional outcomes. Both studies focused on chronic neck pain and demonstrated functional improvements on a standardized self-report measure of neck functioning during or immediately following the VR exposure, with 1 study [10] suggesting continued self-reported functional gains at a 3-month follow-up.

Trost et al [9] provided a timely overview of the existing research on VR applications for pain. The authors highlight the *3 pillars of VR*: presence, immersion, and interactivity, which vary in importance depending on the pain context (acute, experimental to examine mediators or moderators, or chronic). As noted by Trost et al [9], presence refers to the subjective experience of being in one environment while being physically situated in another. Immersion refers to the level of absorption a user experiences in the virtual world, and interactivity refers to the extent to which the user can manipulate the virtual environment. These authors provide a useful heuristic model that outlines technical VR configuration factors (eg, hepatic feedback), user experiential factors (eg, presence), and pain targets (eg, cognitive behavioral therapy skills) that converge to influence outcomes (eg, pain intensity) [9]. Importantly, these dimensions may vary based on the goals of the VR therapy context.

Many chronic pain studies have made the pillars of presence or embodiment and interactivity central to the target. Examples include studies of phantom limb pain [12,13] or spinal cord injury [14,15], with VR potentially amplifying the neuromodulatory effects of movement therapies. Less emphasized in the context of chronic pain is the pillar of immersion, given that distraction is less central in chronic pain management. For example, Villiger et al [16] used 16 to 20 sessions of VR to deliver intensive individual muscle training in patients undergoing neurorehabilitation for spinal cord injuries. The results showed both subjective and objective physical function improvements, with gains remaining stable at 12 and 16 weeks postintervention. Trost et al [17,18] pioneered the use of VR to deliver graded-exposure therapy targeting pain-related fear and disability in adults with chronic pain, recognizing the power of VR to facilitate pain-related movement in the presence of fear and behavioral avoidance. In addition to the recent work in this area specific to chronic pain, we can draw upon more robust literature on the use of VR for exposure-based treatments of other conditions. Several systematic reviews and meta-analyses of virtual reality–based exposure therapy (VRET) for anxiety disorders have shown that VRET is equal or superior to the gold standard of in vivo exposure for anxiety reduction [19-23].

Overall, there is support for using VR and AR as a tool to reduce perceptions of pain intensity in the context of procedural pain, including evidence of effects for children and adolescents. However, a recent review underscores the need for large, well-designed trials to fully evaluate the effects of VR on acute pain in children because of the variability and weaknesses in the study methodology to date [6]. There is growing evidence of the utility of VR and AR for reducing the intensity of chronic pain in the short term and emerging support for using VR in physical rehabilitation and to reduce the fear of pain through exposure-based paradigms in adult populations. The intersection of these areas—that is, the use of VR and AR technology to achieve multiple benefits (reduction of pain, disability, and fear or behavioral avoidance) over a longer period in the context of pediatric chronic pain rehabilitative treatment—is only emerging. To date, published work specifically focused on VR for pediatric chronic pain mainly includes small pilot studies, but these have demonstrated support for using VR to augment established treatments for pediatric chronic pain, including mirror visual feedback therapy for complex regional pain syndrome [24] and biofeedback for pediatric headache [25], with outcomes suggesting increased tolerance of rehabilitative therapies, reduced pain, and improved function and quality of life. Given the increasing accessibility of VR and AR interventions [26], their particular fit for pediatric populations, and their ability to complement and enhance standard approaches for the treatment of complex pediatric pain conditions [27], this is an important and timely area of innovation in our field. As new applications for VR and AR emerge, we need guidelines and best practices to inform the design and evaluation of new potential interventions.

### Objectives

To review the current state of the field of VR for the treatment of chronic pediatric pain and to elucidate important directions for future research and innovation efforts, we convened a working group of experts across relevant domains to share lessons learned through their early work in this area and to generate consensus-backed recommendations for advancing the field. Herein, we provide guidance from the newly formed INOVATE-Pain collaborative (Interdisciplinary Network on Virtual and Augmented Technologies for Pain) for key domains: (1) describing models of academic-clinical-industry partnerships in the development and dissemination of immersive pain intervention approaches; (2) outlining current and future opportunities and challenges for evidence-based innovation, with an emphasis on collaboration across diverse sites, settings, and domains of expertise; and (3) identifying best practices in research on the use of VR and AR in children and adolescents with chronic pain, including recommendations for measuring meaningful outcomes of VR and AR-based interventions.

## Methods

### Participants

The INOVATE-Pain collaborative includes current contributors to science in the field of immersive interventions for pediatric pain rehabilitation, including clinicians, clinical researchers, neuroscientists, and VR software engineers. Potential participants were identified based on their involvement in ongoing work in the realm of VR for pediatric chronic pain treatment. Those first approached by the meeting organizer and chair (DL) were asked to suggest other participants who could represent different perspectives and experiences. Participants included all authors from the following institutions and organizations: Stanford Children’s Health network, the Stanford Childhood Anxiety Reduction through Innovation and Technology (CHARIOT) program, Boston Children’s Hospital (BCH), Cincinnati Children’s Hospital Medical Center (CCHMC), The Hospital for Sick Children (SickKids) in Toronto, University of Southern California or Children’s Hospital of Los Angeles (CHLA), Stanford Virtual Human Interaction Lab, and Mighty Immersion, Inc (a company focused on VR device management tools). This was the first meeting of this group. Follow-up meetings were planned but have not been executed because of the COVID-19 pandemic. The group continues to hold monthly Zoom meetings to continue the work that began at the initial meeting. This meeting was supported by The Mayday Fund. A representative from The Mayday Fund was invited to attend the meeting to provide perspective on the funding landscape and report to The Mayday Fund board on the proposed strategic directions of the INOVATE-Pain collaborative.

### Setting

The meeting took place over the course of 2 days (January 22-23, 2020) in Palo Alto, California.

### Procedures

The impetus for the INOVATE-Pain consensus meeting arose through The Mayday Fund’s efforts to connect individuals engaged in funded research on VR for chronic pain with those seeking funding for proposed projects in this area in the hope of promoting greater collaboration and standardization in the field. The meeting convened with a structured agenda (Multimedia Appendix 1) and planned deliverables for a consortium focused on VR and AR innovations in pediatric pain rehabilitation. Briefly, the meeting began with an overview of the state of the field and moved to reports from each participating academic medical center on their clinical and research-based uses of VR in pediatric chronic pain, followed by a discussion of new technological developments from our industry partner (LW) and his clinical collaborators. Group members representing the funding world and laboratories exploring the use of VR technology in adult populations shared relevant insights. This was followed by a discussion of best practices, with particular emphasis on what outcomes are most relevant to assess when evaluating pediatric chronic pain VR and AR interventions and what tools would be best for such assessments. The second day focused on arriving at a consensus on important new directions in the field and models of collaboration that bring clinicians, researchers, and industry partners together in ways that are mutually beneficial, nonexploitative, and hold patient care as a shared primary goal. Time was devoted to developing INOVATE-Pain’s mission and vision statement, along with strategic goals focused on what our collaborative can offer the field. Finally, we outlined plans for moving forward with actionable, collaborative projects and outputs. Detailed notes were recorded throughout the meetings by the nonparticipants.

The meeting relied on consensus decision making in an open discussion format. To begin, we drew on case examples of current and potential projects overseen by group members to develop a shared understanding of where the field stands and to identify gaps. During discussions, the chair and cochair (LS) kept time limits and reflected themes and major points to the group to ensure that members were in agreement on how these were conceptualized. We then engaged in structured brainstorming sessions derived from ideation methods in design thinking, an approach now being applied to health care innovation and education [28]. We undertook this process for several of our key agenda topics, including identifying important outcome domains to assess in VR studies and developing our collaborative’s mission, vision, and strategic goals. Given the group’s relatively small size, discussions and brainstorming exercises were held with the full group. We adhered to design-thinking brainstorm rules (ie, time limit, stay on topic, defer judgment, encourage wild ideas, aim for quantity, build on each other’s ideas, be visual, and one conversation at a time) utilizing a whiteboard and unlimited Post-its. Following the ideation phase, we grouped similar ideas and labeled these larger constructs, which ultimately became the major themes we discuss in our results (see Multimedia Appendix 2 for this exercise’s visual depiction).

## Results

### Describing Models of Collaborative Partnerships to Advance VR Development, Implementation, and Evaluation

As a first step in identifying successful collaboration models, meeting attendees illustrated ways in which such partnerships have succeeded in their own settings to date.

#### The Stanford CHARIOT Program

The CHARIOT program has led the field in clinical applications and innovations in VR for pediatric procedural anxiety and pain. Having used VR in more than 5000 patients since 2017, the scope of impact and dissemination of VR software tools across 25 institutions worldwide reflects a motivation to rapidly equip hospitals with VR tools for patient care, particularly in the realms of procedural discomfort and anxiety. In addition to the direct distribution of content to these hospitals, the software developed by Stanford CHARIOT has been included on hospital VR platforms used by more than 200 hospitals and clinics in the United States.

#### Stanford Pediatric Pain Rehabilitation

Since 2018, the Stanford Children’s Health Pediatric Rehabilitation Program (PReP) for youth with chronic pain has used VR interventions during physical therapy, occupational therapy, and individual psychology sessions. The program obtained a dedicated VR therapy room for use during the PReP treatment sessions. In collaboration with the Stanford CHARIOT program and through support from The Mayday Fund, a VR platform called Fruity Feet was developed with the PReP interdisciplinary team. This platform was created through an iterative process from PReP provider and patient feedback for improvements and modifications. The overall goal was to improve function and reduce the fear of pain. The initial implementation of Fruity Feet VR tested the acceptability and feasibility of facilitating increased upper and lower extremity engagement [29]. This platform was simultaneously tested with an inpatient population during physical therapy sessions with large effects on movement observed in VR compared with the standard of care physical therapy (Caruso et al, unpublished data, August 2019).

In response to the COVID-19 pandemic, this work involves pilot testing of at-home VR for pain rehabilitation. With PReP operating in a telehealth format for all treatment interventions, youth in PReP were provided with VR equipment for home use during telehealth sessions with providers and independently for their daily home exercise plan. Preliminary data for the at-home VR intervention are forthcoming, but the initial interview feedback is positive.

#### Cincinnati Children’s Hospital Medical Center

Since 2018, the use of VR in patients with pain at CCHMC has followed a -pronged approach: a clinical research arm for managing acute postoperative pain and a clinical application arm for patients with chronic pain undergoing inpatient rehabilitation. Our team has conducted 2 pilot studies in children with moderate to severe pain after surgery, one of which used distraction-based virtual reality (VR-D) and guided-relaxation virtual reality (VR-GR) using the Mindful Aurora app [30-32]. This pilot study demonstrated that a single, postoperative session of both VR-D and VR-GR was associated with small changes in pain and anxiety lasting up to 30 minutes. This pilot study supports the implementation of VR therapy in managing acute postoperative pain in children and the need for future studies. The CCHMC team worked with Stanford CHARIOT to use VR programs appropriate for pediatric patients. This partnership has afforded the clinical team access to VR content that is highly engaging and motivating for children and adolescents.

Going forward, the VR team continues to explore engaging and innovative ways of using VR for pain management. By adding VR to postoperative pain management, we hope to augment our primary strategy of multimodal analgesia by increasing patient motivation and engagement in alternative therapies (eg, distraction and relaxation). Plans are in place to continue the implementation of VR into chronic pain rehabilitation with an emphasis on increasing social interaction in group settings through cooperative VR activities and improving movement and body awareness by incorporating VR into physical and occupational therapy.

#### The Hospital for Sick Children

Clinicians and researchers have been implementing VR at SickKids over the past 8 years in diagnostic imaging and emergency departments for intravenous insertions [33], perioperative services to prepare children for anesthesia during the perioperative period, and for subcutaneous port-a-cath access in oncology (Aqua KindVR) [34,35]. In a single-site pilot randomized controlled trial comparing VR (peaceful underwater gamified environment) with iPad (Apple Inc; underwater video with headphones) for oncology procedures, nurses, parents, and children reported the interventions in both groups to be acceptable, with the VR participants reporting significantly higher immersion, an underassessed outcome metric in studies of VR for pediatric pain. In addition, there were trends toward reports of less pain and distress during procedures in the VR group compared with the iPad group [35].

More recently, the SickKids Chronic Pain Clinic rehabilitation team used the Fruity Feet program developed through collaboration with Stanford CHARIOT in a quality improvement (QI) project to implement and evaluate the hospital’s rehabilitation department. The QI project involved interviews with 10 adolescents and their physical therapists (PT) and found that participants reported a high acceptance of the program, high immersion and high satisfaction, no adverse events, and lower pain scores after using VR. PTs also reported that VR was easy to use and feasible to implement in the pediatric rehabilitation setting. Given the success and clinical need, SickKids has successfully advocated for a dedicated child life specialist to help with the clinical implementation of VR coupled with a programmatic approach to rolling out new interventions using evidence-based methods.

#### Boston Children’s Hospital

With support from The Mayday Fund, the BCH has played a central role in developing the INOVATE-Pain network. This partnership has afforded BCH with opportunities to gain VR expertise from initiatives undertaken by the leaders of the pediatric pain VR field. In addition, this collaboration has provided BCH with opportunities to creatively explore and expand the utilization of VR in the treatment of pediatric chronic pain and to plan research initiatives to evaluate these novel applications.

Since 2010, the BCH’s intensive pain rehabilitation day program has used a basic interactive gaming platform, Xbox Kinect (Microsoft Corporation), to engage youth with chronic pain in their daily treatment. Given this technology’s success and popularity, BCH is actively seeking the integration of newly released VR into their pain rehabilitation program. Initial efforts in this process focus on replicating the success established at Stanford, SickKids, and CCHMC in using VR-based physical therapy, occupational therapy, and psychological interventions in the context of our intensive pain rehabilitation day hospital program. In addition, the BCH team is leading the effort in the development of a novel, immersive VR-based intervention targeting successful school reentry in the context of intensive pain rehabilitation. These innovative efforts have successfully garnered the philanthropic support needed to bring them to fruition.

#### Children’s Hospital of Los Angeles

Clinician-scientists at CHLA have been exploring the use of VR for acute pain management for more than 20 years, pioneering its use with children for routine painful procedures [8,36-38]. Since its inception, the BioBehavioral Pain Lab at CHLA has partnered with academic institutions, technology companies, software developers, and other VR collaborators to maximally leverage content expertise in health care, technology advancements, and input from clinician-scientists to design and implement rigorous VR clinical trials to scientifically investigate the feasibility, usability, and efficacy of VR for managing pain with routine medical procedures. Early and recent efforts with technological advancements have fundamentally changed clinical service lines at CHLA in phlebotomy, where children coming for outpatient phlebotomy can now request VR as a standard of clinical care for blood draws. Recently, VR clinical research has focused on VR home-based systems. As virtual care has been catapulted by telehealth, home-based VR has become critical for ongoing VR investigations.

### Partnerships in Action: The Fruity Feet Experience

**Figure 1:**
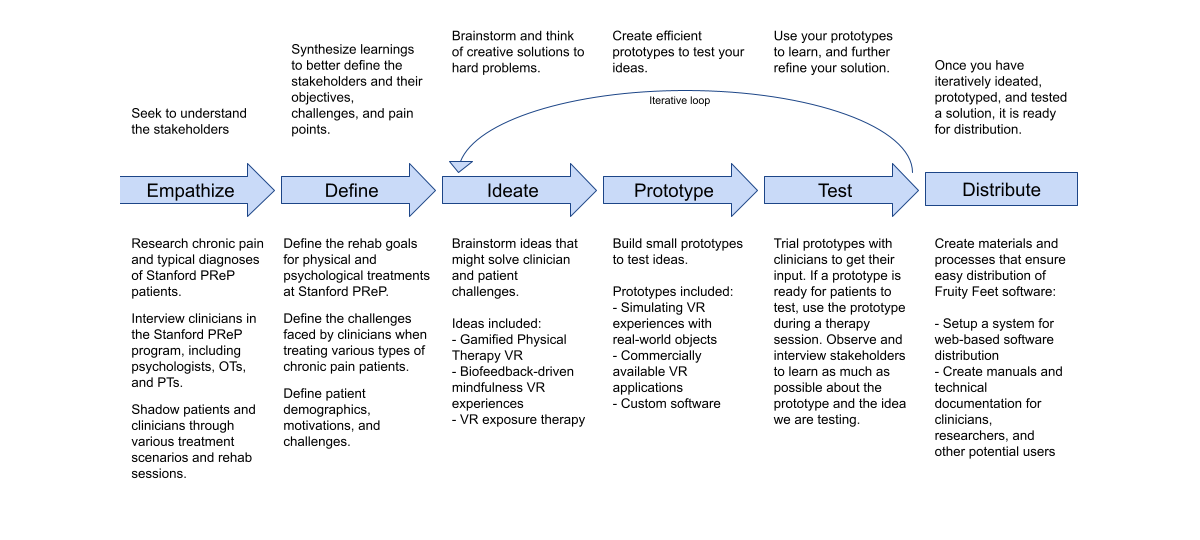
Fruity Feet/Design Thinking Development. PTs: physical therapists; OTs: occupational therapists; VR: virtual reality.

Leveraging our collective experiences thus far, a key deliverable of the meeting was to build from these experiences to define optimal collaborative models to bring clinicians, researchers, industry partners, and funders together in ways that are mutually beneficial, nonexploitative, and hold patient care as a shared primary goal. The industry partner, Luke Wilson of Mighty Immersion Inc [39], provided an overview of the design-thinking framework used to build the VR program developed specifically for pediatric pain rehabilitation treatment centers, *Fruity Feet,* which is currently being implemented at Stanford, CCHMC, and SickKids (Figure 1). In addition to targeted software experiences, we identified additional tools crucial to the successful implementation of VR interventions in patient care settings. First, *device management tools* can facilitate updating all of an institution’s VR tools, track their location in busy clinical settings, and remotely manage devices as needed. Second, *companion applications* enable controlling the VR experience from a second device (eg, provider’s phone), thus tweaking the therapy to fit the patient and therapeutic target. Third, *analytics capture tools* collect movement data, create playlists for VR protocols, and display real-time patient movement. Although seamless integration is the goal, having a technically knowledgeable team member is critical to reducing the clinician time burden and ease of implementation. Industry partners can provide valuable training to develop clinician champions who can oversee VR integration in clinical settings.

### Defining a Mission and Vision for INOVATE-Pain

**Figure 2:**
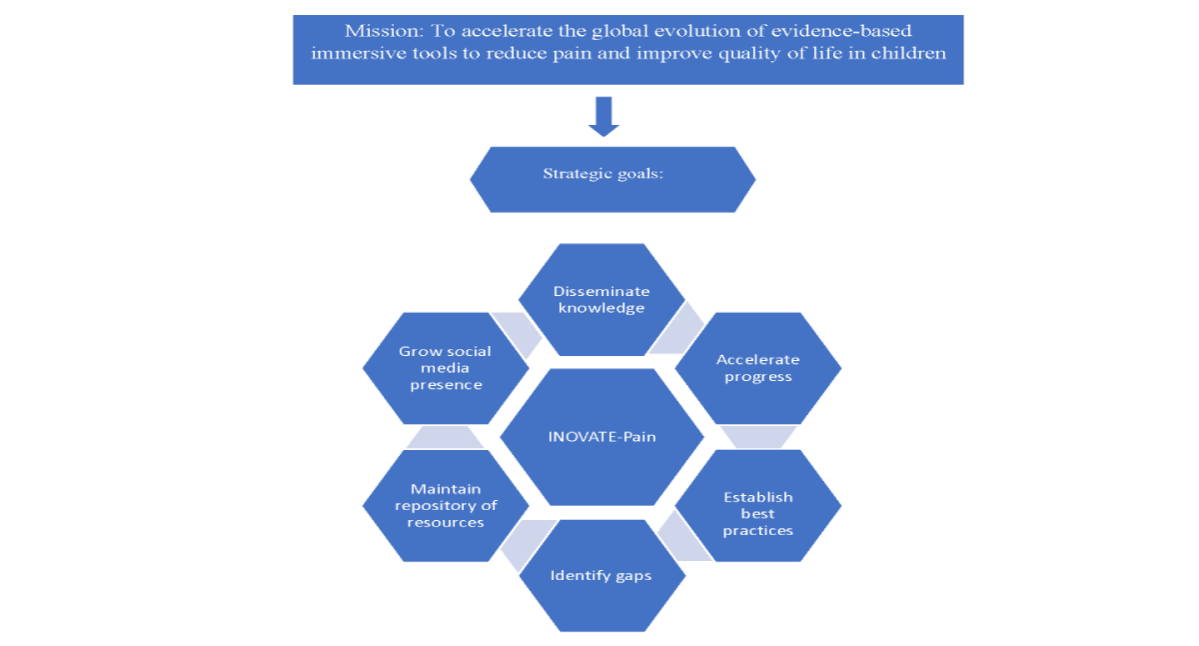
Mission and strategic goals of the INOVATE-Pain (Interdisciplinary Network on Virtual and Augmented Technologies for Pain management) consortium.

Building off this discussion of successful industry-academic partnership, the mission statement, vision, and strategic goals for the INOVATE-Pain collaborative were defined through design-thinking exercises and a consensus process. In formulating our mission, vision, and goals, we sought to differentiate our collaborative from existing programs by asking what we could add to the current efforts in the field. For example, how did we differ from the Stanford CHARIOT program, which uses VR in a broader range of populations and emphasizes the global dissemination of technology? Did we want INOVATE-Pain to be a marketplace for ideas, a repository of resources, and/or an established multisite consortium for new research projects? Through discussion, we identified a strong commitment to fostering and facilitating research and evaluation, given the importance of building an evidence base to support the use of VR interventions. As previously noted, a design-thinking process enabled us to identify the themes that were most widely endorsed by the group as important to our mission and vision, and further discussion of these themes led to full consensus. The mission and vision of accelerating the global evolution of evidence-based immersive tools to reduce pain, reduce disability, and improve children’s quality of life are supported by our strategic goals (Figure 2).

### Outlining Current and Future Challenges and Opportunities in the Field

A second key goal of the meeting was to identify both challenges and opportunities to move the field forward, along with ways that a collaborative network such as INOVATE-Pain could serve to capitalize on the opportunities and surmount the challenges. The 4 major factors were identified as follows: (1) industry versus academic approaches to the evaluation process, (2) discrepant resources and endpoints across academic, clinical, and industry collaborators, (3) proliferation of technology products and companies, and (4) adoption of VR in research and clinical applications.

#### Industry Versus Academic Approaches to the Evaluation Process

The importance of interdisciplinary, multi-institutional, and cross-contextual (industry, academic, and clinical) collaborations toward developing VR software and clinical investigations cannot be understated. However, there are challenges inherent in aligning approaches to the development and evaluation of products in each of these contexts. Navigating logistical, regulatory, and intellectual considerations is a challenge when collaborating with similar institutions (eg, academia), and they are amplified when hospitals and universities attempt to work with industry and commercial partners. Common hurdles that arise include the differing pace of development, the need to navigate multiple regulatory systems, and distinctions in the desired scope or target of the intervention. Start-ups and industries tend to move very quickly with innovation efforts and face fewer regulatory barriers than academic medicine. Large academic and clinical institutions have many layers of regulation, such as intellectual property (IP) considerations when developing products and licensing and billing issues when delivering these interventions in the clinical setting. This raises particular challenges in the realm of digital health and emerging technologies. After a rigorous randomized clinical trial is funded, institutional review is granted, and data are collected and analyzed (a course of action often measured in years), the technology under investigation may be rendered obsolete. However, opportunities exist within these challenges when these 2 approaches can work in a complementary fashion to pursue more nimble approaches to evaluation and dissemination. For example, clinical settings can provide real-world testing grounds and patient populations that are difficult for the industry to access alone. Meanwhile, performing some of the product development in the industry setting may allow the process to move more quickly than projects housed entirely within the academic environment.

In addition to working out issues of project pace and addressing regulatory requirements, partners may differ in their views of project scope. There are advantages and disadvantages to developing interventions tailored to a specific clinical population versus creating widely applicable tools, but potentially less targeted to a particular population (eg, disease condition and age group). This may be particularly true of VR, where there is a huge range of potential software applications and a variety of potential mechanisms of effectiveness that can be harnessed. There is tremendous opportunity to be leveraged when industry and academic partners come together early in the design process so that these advantages and disadvantages can be considered together and interventions can be designed to fit real-world clinical needs, rather than trying to retrofit existing products to use with specific patient populations. Fruity Feet is an excellent example of a collaboration that capitalized on the collaborative process to develop a project that held broad enough appeal to be useful in multiple contexts while also meeting specific needs of children with chronic extremity pain undergoing rehabilitative pain treatment.

#### Academic, Clinical, and Industry Collaborators May Have Different Resources and Endpoints

Related to, but in some ways separate from, the challenges around evaluation design, resources are a major point of negotiation in developing productive partnerships for VR innovation. Resource sharing between collaborators can offer huge benefits in evaluating new interventions, but resource sharing can also multiply hurdles. Given the obligations one assumes when undertaking this type of endeavor and the time and effort required to succeed, it is critical for academic partners to enter carefully into these partnerships to avoid becoming an open testing ground for too many industry products.

For academic and clinical researchers, opportunities lie in the fact that industry partners can bring specific financial benefits to fuel the research endeavor, with tech-savvy personnel and computer science skills exceeding those typically found within academia. In turn, academic partners bring expertise and access to some funding opportunities (eg, National Institutes of Health [NIH] Small Business Innovation Research and similar mechanisms, seed funding to pilot new ideas), along with the ability to gather data for extra- or intramural grant applications, culminating in peer-review publications contributing to the evidence base in VR. Other benefits to academic partners include the possibility of IP or equity in a start-up that could also contribute to academic success or contribute to building a financially secure lab for ongoing research and future collaborations. The industry tends to focus on validating their product through institutional acceptance and the association with large prestigious medical and/or university settings. Conversely, some industry partners want to engage with content expertise to iterate and improve their products to increase efficacy and impact. Ideally, similar-minded academic-industry collaborations focus on creating an ecologically sound product that delivers on the idea that technology can assist in solving complicated health care problems.

#### Proliferation of Technology Products and Companies

The major challenge in the vast immersive technology marketplace is finding effective products that arise from sound research-based development. Over time, the cost of VR hardware has significantly reduced. Once an experimental scientific *ivory tower* effort with limited clinical application, it has evolved into affordable, off-the-shelf products that operate with minimum components to increase ease of use. The accessibility of VR products provides new opportunities for integrating these interventions into clinical care. VR hardware has become highly sophisticated and affordable such that most clinician-scientists can now afford to add it to their toolkit or even work with equipment owned by patients. In addition, as the industry expanded, the inventory has grown from very few available virtual experiences (VEs) to hundreds. Even so, very few VEs have been scientifically well established as gold standard interventions for clinical problems.

#### Adoption of VR in Research and Clinical Applications

Although there are typical barriers to the adoption of new technologies in pediatric health care, VR research and adoption are particularly challenging. First, given the lack of VR safety research in pediatrics, most hardware manufacturers do not recommend VR for children before adolescence. This can further delay institutional review board approval and require additional safety monitoring that is not required with widely accepted hardware, such as tablets. Second, given the typical age of users, the headsets often require custom modifications to appropriately fit children’s head circumference to facilitate such safety and efficacy research. Third, because VR is applied directly to the patient, near the nose and mouth, infection control practices require close attention. Many consumers facing VR products include cloth straps and face pads, which present challenges to sanitation among patients. Finally, although reduced costs have made VR headsets more affordable, they are still a relatively costly adjunct given the lack of reimbursement offered by insurance for their utilization. In the future, appropriate reimbursements will mitigate the cost of implementation.

### Define and Refine Best Practices

The final major goal of our consensus meeting was to begin to develop a set of best practices for conducting methodologically sound research in VR in the context of pediatric chronic pain treatment. The best practices for VR and chronic pain management have not yet been established. Determining best practices is complex, as this academic-industry collaboration has many facets that include but are not limited to hardware products; software products; building an evidence base; and incorporating heterogeneous disease processes; and age, gender, linguistic, and cultural considerations. This section addresses 4 primary areas, recognizing that each area requires specific customizability for the targeted project: (1) assembling the right team, (2) developing or applying the best technology for the target project or population, (3) designing and executing a sound research design and methodology, and (4) disseminating the collaborative work process and study findings.

#### Assembling the Right Team

This is not an easy task, as industry start-ups are often quite eager to partner with academic VR leaders and to gain access to clinical populations to pilot and test their VR experiences. It is critical as a clinician-scientist to thoroughly vet potential partners. In these initial meetings, it is also critical to *right-size* the project and the timeline, that is, determine goals and benchmarks that are agreeable to all partners and appropriate to the desired outcomes. Aligning project scope, timeline, and financial concerns; assembling a team that includes all necessary domains of expertise; and clearly defining each team member’s role are vital preliminary steps to determining the *match* between academia and industry (Textbox 1)*.*

**Guidelines for establishing positive academic-industry partnerships in virtual reality innovation.**

Partner assessment

- What is the reputation and track record of the industry partner?
- Are they a for-profit or nonprofit entity?
- What is their understanding of the research process?
- What are their expectations for what the academic or clinical partner(s) will provide?

Project validation

- What is the project scope?
- What is the expected timeline for deliverables?
- What are the deliverables or intended outcomes?

Understanding funding structure

- Who underwrites what aspects of the project, both in terms of money and effort?
- Is work done for compensation or in-kind arrangement?
- Discuss intellectual property arrangements—include academic or hospital legal representatives

#### Developing or Applying the Best Technology for the Targeted Project or Population

It is essential to carefully define the target population in the early phases of designing a VR and AR-based intervention. There are advantages and disadvantages to developing highly tailored products. On the one hand, clinical utility is paramount, and having an intervention that clearly targets a specific clinical problem increases the effectiveness of the intervention. On the other hand, the time and effort needed to create and evaluate such interventions steer away from creating products that appeal to a broader population in terms of clinical condition, age, and gender. A product that is not sufficiently specific in its intended application may be more challenging to validate through research trials. Ideally, the goal should be to create a suite of tools with options for customizing the experience to achieve a balance between specificity and broad application to a range of patients who can benefit from the intervention.

#### Designing and Executing the Right Research Design and Methodology

After establishing a partnership and aligning the direction and goals of the collaboration, a well-designed study to evaluate the project is vital. As with any clinical research, the goals, available sample, resources, and timeline should drive the study design. Studies to evaluate VR-based interventions can take a variety of scientifically rigorous forms before or in lieu of traditional clinical trials. Depending on the scope and goals, feasibility or usability studies, QI studies to evaluate clinical implementation, pilot studies to inform more definitive trials, and adaptive trial designs to reduce time to results may be appropriate. Ultimately, the use of rigorous design, methods, and standardized patient-reported outcomes will be critical to both the academic and the start-up and industry. The use of flexible and agnostic technology is critical for testing new software programs and moving toward disseminating new information and evidence-based results. It is necessary to set realistic shared visions, missions and objectives and to develop task-oriented timelines with deliverables. There is a need to move away from the traditional, cumbersome randomized controlled trial approach to evaluating VR interventions and toward more adaptive and efficient evaluation methods [40].

A critical element of research design is the thoughtful selection of measures to assess processes and outcomes. To date, there is no clear consensus on how to evaluate a VR experience appropriately or how to tailor the evaluation to the specific context of the intervention. As described earlier, immersion may be central in the context of acute pain distraction, whereas interactivity is paramount for pain rehabilitation and exposure-based VR protocols. Unfortunately, even in the VR field more broadly, there are no gold standard measures of embodiment, presence, and immersion, and more research is needed (see Multimedia Appendix 3 for an example measure of some of these constructs used in pediatric research). In clinical settings, it may also be important to assess provider satisfaction and feasibility in terms of how the intervention fits into the overall workload and workflow. Failure to understand and address these aspects can lead to lack of buy-in from clinical partners (see Multimedia Appendix 4 for an example measure of these constructs).

Although the factors that influence outcomes may vary, the outcomes of interest should remain uniform and consistent across studies and clinical contexts. On the basis of the discussion and consensus at our meeting, we present recommended domains of measurement to evaluate VR interventions for pediatric chronic pain (Textbox 2). Some of these domains have been deemed critical for pediatric chronic pain within the NIH common data elements (CDEs [41]). These CDEs include pain intensity, pain interference, functioning, pain catastrophizing, and treatment satisfaction [41]. Additional general domains considered particularly important for VR include affect and fear of pain or movement. Moreover, we highlight the VR-specific metrics to consider. These include physical movement, energy expenditure, physiology, and immersion. In the context of pain rehabilitation, increasing movement is a highly relevant outcome that can be measured in real time [29]. An added consideration is the frequency of assessment, as it is likely that data collection may range from continual, session-contingent, daily, or milestone-based (eg, start of treatment and discharge). Ideally, a thorough measurement of an intervention will consist of a combination of these measurement timelines. For example, a study could include objective, continuously collected measures of physical activity and movement metrics cataloged during VR sessions, brief questionnaires deployed daily to capture gradual changes and enable single case experimental design analyses [42], and a longer battery of questionnaires completed at specified time points (eg, baseline and 3-month follow-up) to assess changes over time from repeated VR exposure.

**Recommended domains of measurement to evaluate virtual reality interventions for chronic pain.**

Pediatric chronic pain

- Pain intensity (common data element for pediatric chronic pain from the National Institutes of Health recommendations [41]) and unpleasantness
  Visual Analogue Scale [43]
  Numerical Rating Scale [44]
- Pain interference (common data element for pediatric chronic pain from the National Institutes of Health recommendations [41])
  Brief Pain Inventory [45]
  PROMIS-Pain Interference [46]
- Functioning
  Functional Disability Inventory [47]
  Lower Extremity Function Scale [48]
  Upper Extremity Functional Index [49]
  Canadian Occupational Performance Measure [50]
- Fear of pain and movement
  Fear of Pain Questionnaire-Short Form [51]
  Tampa Scale of Kinesiophobia [52]
- Pain catastrophizing (common data element for pediatric chronic pain from the National Institutes of Health recommendations [41])
  Pain Catastrophizing Scale [53]
- Affect
  Positive and Negative Affect Schedule 10 item [54]
  Childhood Anxiety Sensitivity Index [55]
- Satisfaction with treatment (common data element for pediatric chronic pain from the National Institutes of Health recommendations [41])
  Patient Global Impression of Change [56]

Virtual reality specific

- Physical movement
  Motion capture [57]
  Actigraphy [58]
  Wearables
- Energy expenditure
  YUR Fit app
  Wearables
- Physiology
  Heart rate
  Galvanic skin response
  Respiration
  Functional magnetic resonance imaging
- Immersion or presence
  Child presence measure (Multimedia Appendix 2)

#### Dissemination of the Academic-Industry Collaborative Work Group and Study Findings

Not all collaborative efforts were the same. From the onset, it is critical to set mutually agreed-upon deliverables. As previously noted, academic goals are often quite different from industry objectives. Some of these discrepancies need to be addressed in the first phase of the academic-industry collaborative, establishing clear goals or deliverables. The framework (*for-profit* or *nonprofit*) can greatly influence the end goal. Ultimately, both the collaborative process and the findings derived from the academic-industry collaborative need to be published in peer-reviewed journals and disseminated widely to promote the innovation and proliferation of evidence-based technology in health care. To that end, healthy, transparent, well-communicated projects can lead to fruitful and rewarding collaborations for the academic and medical partners, industry, patient and their family, and ultimately, society. INOVATE-Pain is working to create a repository for protocols, products, resources, and recommendations to guide study design and clinical implementation that can be openly accessed in an effort to advance dissemination of the work in this field and increase opportunities for collaboration.

## Discussion

### Principal Findings and Next Steps

In an area as rapidly evolving and complex as digital health, there is a need for multisite efforts and cross-disciplinary collaboration to keep pace with emerging technology and develop sophisticated studies that build a sound and useful evidence base. Our consortium brought together expertise in software development, clinical applications, experimental work in VR, child psychology, physical or occupational therapy, experience in navigating IP issues and bringing industry partners into the hospital setting, and funders who provided insight into how projects can be competitive for financial support. This represents a diverse group that does not meet often to think of the challenges and opportunities in the field. Our guidance and recommendations are aimed primarily at academic and clinical partners, and we hope that further work by our group can also provide more guidance targeting best practices on the industry side of these partnerships. Furthermore, given the nascent nature of this area of innovation, our current focus is primarily on research and evaluation of new interventions. However, the ultimate goal is for VR interventions to become an evidence-based, widely adopted routine component of clinical care in pediatric chronic pain treatment.

Work to date highlights tremendous opportunities in this area of digital health innovation, with immense promise for improving the treatment of pediatric chronic pain. However, this remains a challenging field to navigate given the number of outstanding issues regarding how to identify and form productive collaborations across academic, clinical, and industry partners; how to design and obtain resources to support solid research protocols to evaluate potential interventions; and how to bring interventions from conceptualization into clinical use in large, complex institutional settings where they can be accessible to patients with the greatest needs.

We identified several important next steps to advance the field toward our mission of accelerating the global evolution of evidence-based immersive tools to reduce pain and improve the quality of life in children with pain. These include the following:

1. *Developing and maintaining a repository of resources.* We are working to collect and curate protocols, publications, available software products, information on funding mechanisms, and other resources to serve as a clearinghouse for researchers and academically oriented VR experience designers. Vetting information and opportunities and housing this information in a centralized, accessible, web-based location may help to lower the barrier to engaging in this field and better standardize the approach to evaluating new interventions. We plan to work synergistically with other like-minded groups in the field, such as the Invincikids nonprofit consortium [59].
2. *Continue our efforts to establish a gold standard set of outcomes to be measured in pediatric pain VR research and specific recommended measurement tools.* Well-validated measures exist in some of the recommended outcome assessment domains we describe but are notably lacking in domains including presence, immersion, interactivity, impact on clinical workload or workflow, and the subjective experience of both the clinician and patient users. Imminent steps toward this goal include undertaking a Delphi process to determine a minimal core data set for pediatric pain VR research and, where gaps are identified, working to develop tools to fill these gaps.
3. *Offer opportunities for education and connections in the field.* Through training opportunities and symposia, we hope our group can increase exposure to the work that has been done to date, disseminate current best practices, and facilitate connections among additional potential collaborators and industry partners who can bring new energy and ideas to advance the INOVATE-Pain mission.
4. *Work together to advance current projects and launch new interventions in a rigorous, appropriately resourced environment.* For example, we have begun planning a trial to develop and evaluate a VR intervention that exposes patients in pediatric intensive pain rehabilitation treatment to the challenges they face in returning to school settings. This intervention would incorporate physical therapy, occupational therapy, and psychological aspects and would be tailored to the specific fears and barriers each patient identifies, making returning to school such a challenging goal. We look forward to designing this project in the context of the INOVATE-Pain collaborative, where we can seamlessly access the full range of expertise needed to develop this type of patient experience and access to a large patient population by scaling the project up to multisite data collection. This is an example of the goals we can attain through the partnerships we have sought to create in this collaborative. Through specific projects, we also hope to evolve the approach to studying VR interventions in the pediatric pain environment, with the goal of balancing the rigor of traditional clinical trial design with the efficiency and creativity needed to deliver promising VR products and experiences to pediatric chronic pain patients in a timeframe that keeps pace with digital health technology innovation.

### Conclusions

In summary, VR is an exciting and promising digital health tool whose applications for reducing pediatric chronic pain are just emerging. To realize the promise of this realm of innovation, key ingredients for success include productive partnerships among industry, academic, and clinical stakeholders; a uniform set of outcome domains and measures for standardized evaluation; and easy, widespread access to the latest opportunities, tools, and resources. By exploring the current opportunities, challenges, best practices, and important next steps in VR for pediatric chronic pain, the INOVATE-Pain collaborative hopes to promote the creation, rigorous yet efficient evaluation, and dissemination of innovative VR-based interventions to reduce pain and improve quality of life for children.

## Appendix

Multimedia Appendix 1 Meeting agenda.

Multimedia Appendix 2 Visual depiction of ideate brainstorming and consensus building exercise.

Multimedia Appendix 3 Sample measure of child presence in the virtual reality experience.

Multimedia Appendix 4 Sample measure of virtual reality acceptability and impact on workflow in the clinical setting.

## Abbreviations

AR: augmented reality
BCH: Boston Children’s Hospital
CCHMC: Cincinnati Children’s Hospital Medical Center
CDE: common data element
CHARIOT: Childhood Anxiety Reduction through Innovation and Technology
CHLA: Children’s Hospital of Los Angeles
INOVATE-Pain: Interdisciplinary Network on Virtual and Augmented Technologies for Pain management
IP: intellectual property
NIH: National Institutes of Health
PReP: Pediatric Rehabilitation Program
PT: physical therapist
QI: quality improvement
SickKids: The Hospital for Sick Children
VE: virtual experience
VR: virtual reality
VR-D: distraction-based virtual reality
VRET: virtual reality–based exposure therapy
VR-GR: guided-relaxation virtual reality
