# Supplementary material for: Leveraging Virtual Reality and Augmented Reality to Combat Chronic Pain in Youth: Position Paper From the Interdisciplinary Network on Virtual and Augmented Technologies for Pain Management
Source: J Med Internet Res. 2021 Apr 26;23(4):e25916. doi: 10.2196/25916 (PMC8111507; doi:10.2196/25916)
Supplement: Multimedia Appendix 1 [file jmir_v23i4e25916_app1.docx]

Supplement 1.

| INOVATE-Pain Meeting Agenda |
| --- |
| Meeting Details: Palo Alto CA. January 22-23^rd^, 2020  Meeting Chair: Deirdre Logan, PhD, Boston Children’s Hospital  Co-Chair: Laura Simons, PhD, Stanford University |
| Day One:   1. **Overview and Introduction** 2. **Demonstrations of current VR software being used in clinical settings** 3. **State of the Art and Science of VR for Chronic Pain**    1. Summary of the current research    2. Attendee presentations of current VR-related efforts in their clinical settings    3. Technical updates and possibilities for immersive engagement (reported from industry partner Mighty Immersion and Stanford Chariot team)    4. Discussion of current measures of engagement, movement, and outcomes with VR – identifying current gold standards and highlighting gaps to address 4. **Identify current strengths and gaps in the work to date**    1. Identify clinical needs    2. Discussion of best practices in evaluation    3. What outcomes are appropriate to assess and how are they best measured? |
| Day Two:   1. **New Frontiers and Funding for VR in IIPT:**    1. Overview of funding landscape for VR for pain/other relevant subgroups    2. Currently funded work, and funding opportunities    3. Discussion of ethical and productive models of collaboration among clinicians, clinical researchers, and industry partners    4. A test case of a novel VR/AR collaborative project: School re-entry exposure 2. **Vision, mission, strategic goals for the INOVATE-Pain Consortium**    1. Initial brainstorm activity    2. Refining/defining our vision, mission and goals 3. **Consensus discussion to agree on meeting output and action items to move the field forward** |
