# Supplementary figures and images for: Leveraging Virtual Reality and Augmented Reality to Combat Chronic Pain in Youth: Position Paper From the Interdisciplinary Network on Virtual and Augmented Technologies for Pain Management
Source: J Med Internet Res. 2021 Apr 26;23(4):e25916. doi: 10.2196/25916 (PMC8111507; doi:10.2196/25916)

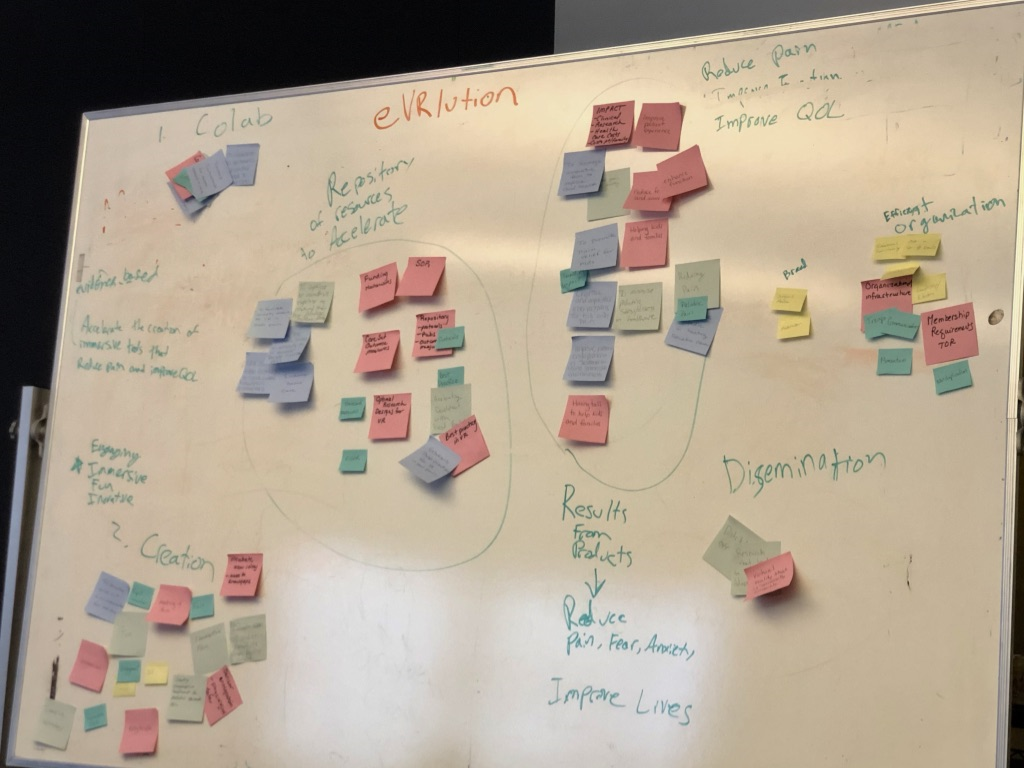

Supplement: Multimedia Appendix 2 [file jmir_v23i4e25916_app2.png]

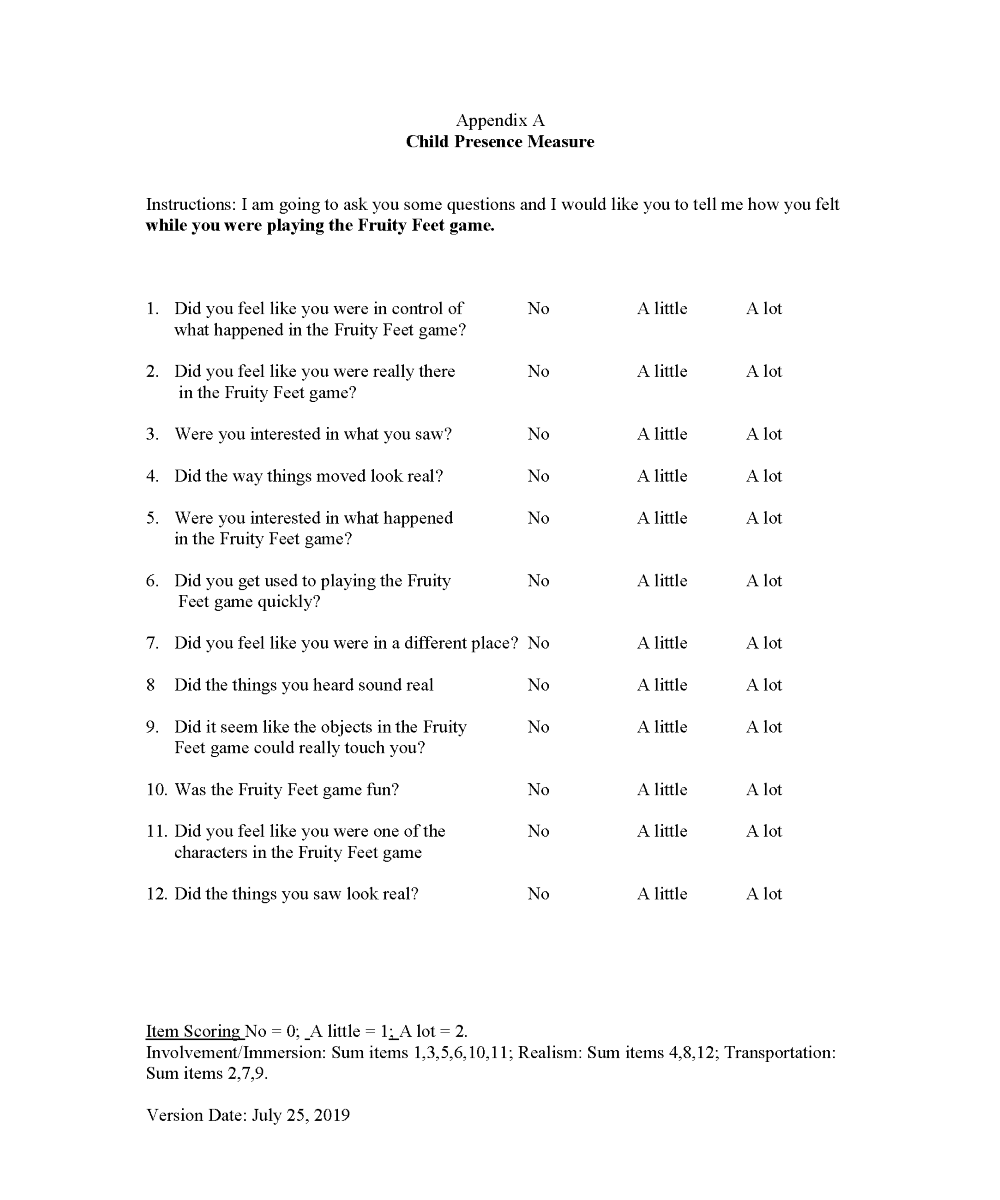

Supplement: Multimedia Appendix 3 [file jmir_v23i4e25916_app3.png]
