# Supplementary material for: Leveraging Virtual Reality and Augmented Reality to Combat Chronic Pain in Youth: Position Paper From the Interdisciplinary Network on Virtual and Augmented Technologies for Pain Management
Source: J Med Internet Res. 2021 Apr 26;23(4):e25916. doi: 10.2196/25916 (PMC8111507; doi:10.2196/25916)
Supplement: Multimedia Appendix 4 [file jmir_v23i4e25916_app4.doc]

Post VR Use Healthcare Team Questionnaire

Number of VR Sessions to date: _____

1. Did the patient/family agree to using VR? y/n (if no is selected, why not?)
2. Did the patient have any contraindications or relative contraindications to VR (select all that apply: None; history or increased risk of headaches/migraines, motion sickness; history or increased risk nausea; currently on sedating medications; age 6 years or younger; history or increased risk of seizures).
3. Which VR modules were used in today's session?
4. Did the patient experience any side effects to VR? (select all that apply)

None; nausea, motion sickness, vomiting, eye strain, family memory formation, injury, other - please describe.

1. Did VR session cause any delays following patient appointment today? Yes/No
2. Duration from start to finish (including set up and take down) of VR session. Time______
3. Any issues with clinic space and equipment – access, double booking, equipment available, equipment in working order? Yes/No. If yes please detail.
4. Is there anything that could be improved in the VR sessions?

# Other comments:

**Clinic staff perspective Usability and Acceptability Focus Group Questions for Clinicians and Clinic Staff:**

**Date:**--------

1. **Clinical flow.** Please describe the impact VR had on clinical work flow (any impact on other patient appointments, any scheduling conflicts, delay of following patient appointment, etc).
2. **Time for VR.** Please describe how long it too for VR sessions in terms of set up, using it with patient and take down and does this time seem reasonable.
3. **Knowledge base and skillset with the technology.** Did you feel prepared to start using the VR (some areas to explore further are ability to troubleshoot with the technology, comfort level, ease of use, confidence). If so what made you feel prepared, if not what would you like to have had in terms of training to make you more prepared to provide this clinical care.
4. **Describe your experiences using the VR technology.** What did you like? What did you dislike? Please describe. How did you approach integrating it into your practice?
5. Were there any benefits to using the VR technology? If so, please describe.
6. Were there any challenges to using the VR technology? If so, please describe.
7. What was the impact on your workflow?
8. If you were going to implement VR into your care practice, how would you do this? Probes: Resources, workflow, environment, etc.
9. Is there anything else about the VR program that we didn't ask you that you would like to provide feedback on?

**Healthcare Team Satisfaction**

**Date:**

| **1. How easy was conducting the session while the VR program was being used?** | | | | |
| --- | --- | --- | --- | --- |
| 0 | 1 | 2 | 3 | 4 |
| very difficult |  |  |  | very easy |
| **2. How much did you enjoy the presence of the VR program during the session?** | | | | |
| 0 | 1 | 2 | 3 | 4 |
| not at all |  |  |  | very much |
| **3. How helpful was the VR program in conducting the session?** | | | | |
| 0 | 1 | 2 | 3 | 4 |
| very unhelpful |  |  |  | very helpful |
| **4. How helpful was the VR program in reducing the child's pain?** | | | | |
| 0 | 1 | 2 | 3 | 4 |
| very unhelpful |  |  |  | very helpful |
| **5. Was the amount of time it took to complete the session using the VR program acceptable?** | | | | |
| 0 | 1 | 2 | 3 | 4 |
| Very unacceptable |  |  |  | Very acceptable |
| **6. How would you rate your overall satisfaction with the VR program?** | | | | |
| 0 | 1 | 2 | 3 | 4 |
| Very dissatisfied |  |  |  | Very satisfied |
| **7. How much did the VR program negatively impact on clinical workflow?** | | | | |
| 0 | 1 | 2 | 3 | 4 |
| Not at all |  |  |  | Completely |
